# The complete genome of *Escherichia* phage Midge

**DOI:** 10.1128/mra.00861-25

**Published:** 2025-10-31

**Authors:** Paige Ripberger, Guadalupe Valencia-Toxqui, Jolene Ramsey

**Affiliations:** 1 Department of Biology, Texas A&M University 14736, College Station, Texas, USA; 2 Center for Phage Technology, Texas A&M University 14736, College Station, Texas, USA; University of Wisconsin-Madison Bacteriology, Madison, Wisconsin, USA

**Keywords:** bacteriophages, genomics, *Escherichia coli*

## Abstract

In this report, we describe the isolation and genomic annotation of *Escherichia* phage Midge. A P2-like myophage, Midge, has a 29,617 bp genome. By nucleotide similarity, Midge is most closely related to Erwinia phage EtG.

## ANNOUNCEMENT

*Escherichia coli* is a commensal bacterium commonly found in the mammalian gut. *E. coli* strain 4s is an equine commensal that was isolated from a longitudinal horse microbiome study (1, 2). In this report, we describe the isolation and genomic annotation of the 4s-infecting myophage, which we called Midge.

Midge was isolated from an enrichment culture of 10 mL of 0.2 μm-filtered freshwater from Finfeather Lake, Bryan, TX (30°38′55.3″N 96°22′26.3″W) and 100  µL of *E. coli* 4s grown aerobically in lysogeny broth at 37°C. The phage was propagated three times by the soft agar overlay method (3). DNA extraction from DNase-treated polyethylene glycol-precipitated lysate used the Norgen Biotek kit (#46800) according to manufacturer recommendations. SeqCoast Genomics (Pittsburgh, PA) performed sequencing using the Illumina DNA Prep tagmentation kit and unique dual indexes on a NextSeq2000 platform (300-cycle flow cell), generating 2×150 bp reads. The 1,785,843 total sequence reads were quality controlled with FastQC (www.bioinformatics.babraham.ac.uk/projects/fastqc), and assembled into a single contig at 109.8-fold coverage using Shovill-SPAdes v1.1.0 with default parameters (4, 5). Contig accuracy and completeness were confirmed by PCR and Sanger sequencing using primers listed in the GenBank record (PV287706.1); no terminal repeats were detected. The Midge genome was re-opened before the portal gene according to convention (6), then annotated using the Phage Galaxy and Apollo interfaces (https://phage.usegalaxy.eu), as described in (7). Structural annotation was performed using GLIMMER v3.02 (8), MetaGeneAnnotator v1.0 (9), and GetORFs v19.1.0.0 (10), while tRNA coding regions were predicted by ARAGORN v19.1.0.0 (11). The function of the called genes was predicted by BLAST v2.10.1 (12), InterProScan v5.59 (13), and TMHMM v2.0 (14). Supporting analysis was performed using the HHpred tool (15). All software used the default settings.

The phage Midge has a double-stranded DNA genome with 29,617 bp and 55% G + C content, similar to the 50% G + C content of its 4s host (Table 1). Analysis of Midge’s genome predicts a total of 47 protein-coding genes, of which 41 were assigned functional predictions . The Midge lysis proteins include a class II holin (GenBank acc. XRM24217), signal-arrest-release endolysin (XRM24218), and a pair of inner (XRM24220) and outer (XRM24221) membrane spanins. Midge shares nucleotide similarities of 75.3% and 52.3% with Erwinia phage EtG (Genbank acc. MF276773) and Salmonella phage FSL SP-004 (NC_021774). Phylogenetic analysis with Virus Intergenomic Distance Calculator (VIRIDIC) (16) suggests classification within the *Peduoviridae*. The presence of structural proteins, specifically the tail sheath protein (XRM24230.1) and the tail tube protein (XRM24231.1) and phylogenetic relationships suggest that Midge is a myophage. Midge is identified as a P2-like phage based on its genomic organization and the structural and functional similarities of 31 proteins, although its genome is smaller than the 33,593 bp of phage P2 (Genbank acc. NC_001895). Although Midge and P2 differ in genome size, their shared features and the adaptability of P2-like phages present opportunities for their use in genome engineering (17).

**TABLE 1:** Genomic characteristics of Escherichia phage Midge

Feature	Value
Genome size (bp)	29,617
GC content (%)	55
Sequence reads	1,785,843
Coverage (fold)	109.8
Completeness (%)	100
Coding sequences	47
Predicted functions assigned	41 (87.2%)
Hypothetical proteins	6 (12.8%)
Genbank accession	PV287706.1

## Data Availability

The genome sequence and associated data for phage Midge were deposited under GenBank accession number PV287706.1, BioProject number PRJNA222858, Sequence Read Archive number SRR32671507, and BioSample number SAMN47326487.
